# ABCA13 dysfunction associated with psychiatric disorders causes impaired cholesterol trafficking

**DOI:** 10.1074/jbc.RA120.015997

**Published:** 2020-12-16

**Authors:** Mitsuhiro Nakato, Naoko Shiranaga, Maiko Tomioka, Hitomi Watanabe, Junko Kurisu, Mineko Kengaku, Naoko Komura, Hiromune Ando, Yasuhisa Kimura, Noriyuki Kioka, Kazumitsu Ueda

**Affiliations:** 1 Division of Applied Life Sciences, Graduate School of Agriculture, Kyoto University, Kyoto, Japan; 2 Institute for Frontier Life and Medical Sciences, Kyoto University, Kyoto, Japan; 3 Institute for Integrated Cell-Material Sciences (WPI-iCeMS), Kyoto University, Kyoto, Japan; 4 Institute for Glyco-core Research (iGCORE), Gifu University, Gifu, Japan; 5 Center for Highly Advanced Integration of Nano and Life Sciences (G-CHAIN), Gifu University, Gifu, Japan

**Keywords:** ABC proteins, ATP binding cassette subfamily A member 13 (ABCA13), psychiatric disorders, cholesterol, gangliosides, prepulse inhibition, endocytic retrograde transport, synaptic vesicle endocytosis, ABCA13, ATP-binding cassette subfamily A member 13, DMEM, Dulbecco's modified Eagle's medium, MβCD, methyl-beta-cyclodextrin, NBDs, nucleotide-binding domains, TMDs, transmembrane domains

## Abstract

ATP-binding cassette subfamily A member 13 (ABCA13) is predicted to be the largest ABC protein, consisting of 5058 amino acids and a long N-terminal region. Mutations in the ABCA13 gene were reported to increase the susceptibility to schizophrenia, bipolar disorder, and major depression. However, little is known about the molecular functions of ABCA13 or how they associate with psychiatric disorders. Here, we examined the biochemical activity of ABCA13 using HEK293 cells transfected with mouse ABCA13. The expression of ABCA13 induced the internalization of cholesterol and gangliosides from the plasma membrane to intracellular vesicles. Cholesterol internalization by ABCA13 required the long N-terminal region and ATP hydrolysis. To examine the physiological roles of ABCA13, we generated Abca13 KO mice using CRISPR/Cas and found that these mice exhibited deficits of prepulse inhibition. Vesicular cholesterol accumulation and synaptic vesicle endocytosis were impaired in primary cultures of Abca13 KO cortical neurons. Furthermore, mutations in ABCA13 gene associated with psychiatric disorders disrupted the protein's subcellular localization and impaired cholesterol trafficking. These findings suggest that ABCA13 accelerates cholesterol internalization by endocytic retrograde transport in neurons and that loss of this function is associated with the pathophysiology of psychiatric disorders.

ATP-binding cassette (ABC) proteins constitute a transporter superfamily that plays important physiological roles in all living organisms (1, 2). ABC proteins couple the energy of ATP binding and hydrolysis to many biological processes such as the translocation of various substrates including lipids, ions, peptides, and xenobiotics (3). Defects in the function and expression of ABC proteins are related to various diseases (1).

ATP-binding cassette subfamily A member 13 (ABCA13) is a transmembrane protein with the typical structure of ABC proteins: two transmembrane domains (TMDs) and two nucleotide-binding domains (NBDs) characterized by Walker A, Walker B, and ABC signature motifs (4). ABCA13 is predicted to be the largest member of the ABC protein family, and the human form includes 5058 amino acid residues and a long N-terminal region. In addition, alternative ABCA13 transcripts and protein lacking the N-terminal region were also reported (5, 6). The calculated molecular masses of full-size and shorter human ABCA13 are about 570 kDa and 260 kDa, respectively. ABCA13 is expressed in the human trachea, testis, bone marrow, brain, and other tissues (4, 5, 7).

ABCA13 belongs to the ABCA subfamily (8). Most proteins in this subfamily are reported to transport lipids (9), including cholesterol and phosphatidylcholine by ABCA1 (10, 11), surfactant lipids by ABCA3 (12, 13, 14, 15), N-retinylidene-phosphatidylethanolamine by ABCA4 (16), phosphatidylcholine and lysophosphatidylcholine by ABCA7 (17, 18), and glucosylceramide by ABCA12 (19). These findings suggest that ABCA13 too transports lipids. However, the function of ABCA13 is unknown.

Interestingly, rare genetic variants of human ABCA13 are related to susceptibility for schizophrenia, bipolar disorder, and major depression (7), but not without controversy. For example, one replication study on some of these rare variants failed to find this relation in an independent sample set (20). A monkey carrying the heterozygous ABCA13 deletion displayed impaired social ability and restricted and repetitive behaviors that are most frequently associated with autism spectrum disorder (21, 22). However, the same monkey had a nonsense mutation in 5-hydroxytryptamine (serotonin) receptor 2C. 5-Hydroxytryptamine receptor 2C has been associated with neuropsychiatric disorders including autism spectrum disorder, leaving it unclear whether the heterozygous ABCA13 deletion caused the autism spectrum disorder-like phenotypes. Additionally, in a *Drosophila* model, knockdown of CG1718, which is homologous to human ABCA13, induced increased social space and abnormal circadian rhythm (23). However, in reality, the amino acid sequence and protein size of CG1718 are more similar to that of ABCA3 than ABCA13.

In this study, to elucidate the molecular functions of ABCA13, we examined the subcellular localization and function of ABCA13 in HEK293 cells transfected with mouse ABCA13. To study the physiological roles of ABCA13, we generated Abca13 KO mice using the clustered regularly interspaced short palindromic repeats/CRISPR-associated proteins (CRISPR/Cas) system and investigated the impact of ABCA13 deletion on behavioral phenotypes. In addition, we examined the intracellular cholesterol distribution and synaptic vesicle endocytosis in Abca13 KO cortical neuronal cultures. We performed ABCA13 mutant analysis to determine the effects of mutations associated with psychiatric disorders on ABCA13 function, finding that abnormal psychological behaviors correlated with impairments in the subcellular localization of and cholesterol trafficking by ABCA13.

## Results

### Full-size ABCA13 (570 kDa) is localized in intracellular vesicles

**Figure 1:**
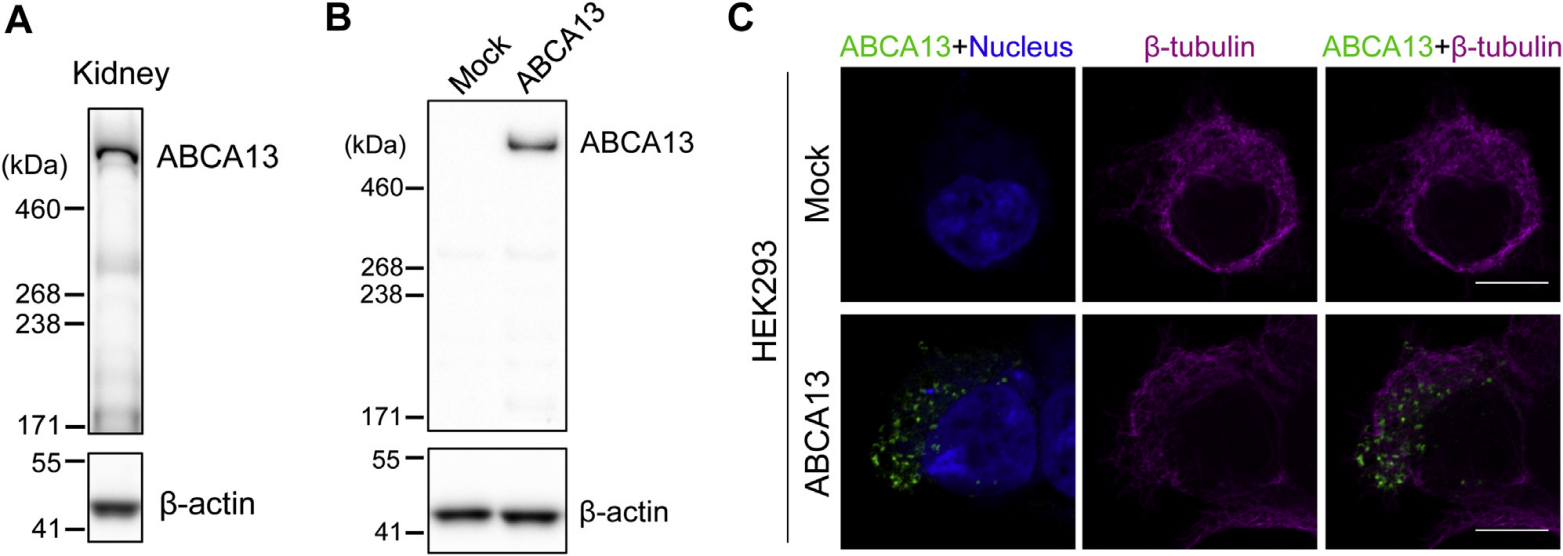
**Full-size ABCA13 is localized in intracellular vesicles.***A*, Western blot analysis of kidneys from WT mice using anti-ABCA13 antibody. β-actin was used as a loading control. *B*, HEK293 cells were transiently transfected with mouse ABCA13. ABCA13 expression was confirmed by western blotting. β-actin was used as a loading control. *C*, cells were immunostained with anti-ABCA13 antibody (*green*) and anti-β-tubulin antibody (*magenta*) after fixation and permeabilization. Nuclei were stained with TOTO-3 (*blue*). Scale bars represent 10 μm.

To determine the size of ABCA13 mainly expressed in mice, western blotting was performed using mouse kidney (Fig. 1*A*), where Abca13 is highly expressed. A major band was mainly detected at >460 kDa, which is consistent with the predicted molecular mass of full-size ABCA13 (570 kDa). This result indicates that ABCA13 is mainly expressed as a protein containing the long N-terminal region in mice. To study the biochemical activity of full-size ABCA13, we examined the protein's subcellular localization. HEK293 cells were transiently transfected with a plasmid expressing full-length mouse ABCA13 (5034 amino acids). Western blot analysis (Fig. 1*B*) showed a band of similar size to ABCA13 expressed *in vivo*. Immunostaining revealed that ABCA13 was localized in intracellular vesicles in HEK293 cells (Fig. 1*C*).

### ABCA13 expression results in cholesterol accumulation in intracellular vesicles

**Figure 2:**
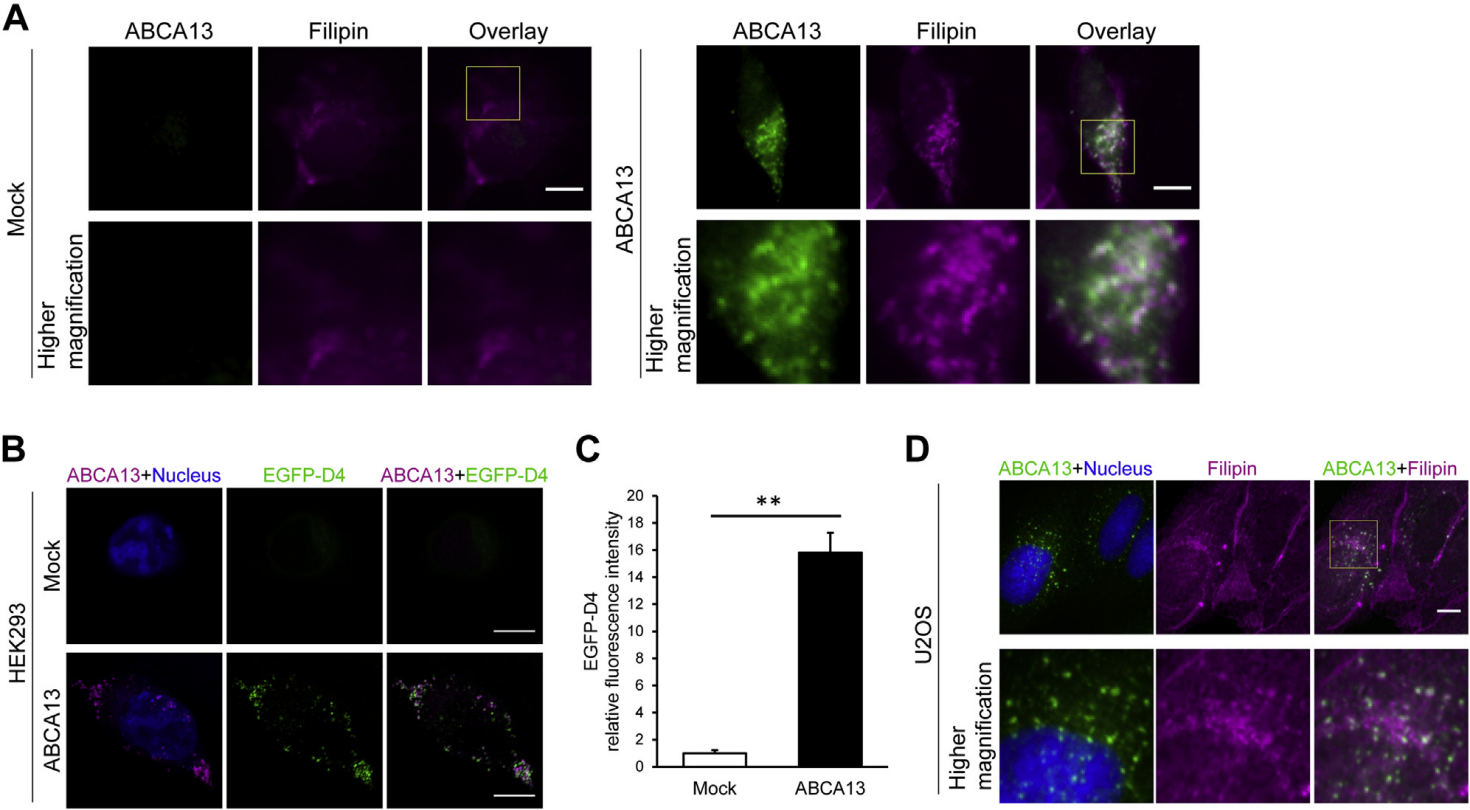
**ABCA13 triggers cholesterol accumulation in intracellular vesicles**. *A*, HEK293 cells transiently transfected with ABCA13 were labeled with anti-ABCA13 antibody (*green*) and the fluorescent cholesterol-binding probe filipin (*magenta*) after fixation and permeabilization. *B*, HEK293 cells transiently transfected with ABCA13 were labeled with anti-ABCA13 antibody (*magenta*) and the fluorescent probe EGFP-D4 targeting cholesterol-rich membrane domains (*green*) after fixation and permeabilization. Nuclei were stained with TOTO-3 (*blue*). *C*, the relative fluorescence intensity of EGFP-D4 signals in individual cells was quantified using ImageJ and shown as means + S.E.M. (mock, n = 8; ABCA13, n = 10). ∗∗*p* < 0.01. *D*, U2OS cells, a human osteosarcoma cell line, were fixed and labeled with anti-ABCA13 antibody (*green*) and filipin (*magenta*). Nuclei were stained with propidium iodide (*blue*). The bottom figures are higher magnification images of the area outlined in *yellow*. Scale bars represent 10 μm.

Many members of the ABCA subfamily play important roles in lipid transport processes (8, 10, 11, 12, 13, 14, 15, 16, 17, 18, 19). To determine whether ABCA13 is involved in lipid trafficking, we compared the intracellular cholesterol distribution in control and ABCA13-expressing HEK293 cells using the fluorescent cholesterol-binding probe filipin (Fig. 2*A*). ABCA13-positive vesicles were colocalized with the fluorescent signals in ABCA13-transfected cells. On the other hand, the intense signals of filipin in vesicles were much less observed in control cells. The fluorescent probe EGFP-D4 was reported to target cholesterol-rich membrane domains (24, 25). Consistently, the vesicles where ABCA13 was detected were also strongly labeled with EGFP-D4 (Fig. 2*B*), but EGFP-D4 staining was hardly observed in control cells. Additionally, quantitative image analysis showed that EGFP-D4 staining was significantly higher in ABCA13-transfected cells compared with mock cells (Fig. 2*C*). These results suggest that transiently expressed ABCA13 causes cholesterol accumulation in vesicles.

To examine whether human endogenous ABCA13 is localized in intracellular vesicles and affects intracellular cholesterol distribution, we used U2OS cells, a human osteosarcoma cell line that expresses endogenous ABCA13 (Fig. S1). As with our HEK293 experiments, in U2OS cells, intracellular vesicles where ABCA13 was detected were strongly stained with filipin (Fig. 2*D*). These results indicate that endogenously expressed human ABCA13 causes cholesterol accumulation in intracellular vesicles (ABCA13-vesicles) like mouse ABCA13.

### ABCA13 accelerates the internalization of cholesterol and gangliosides by endocytic retrograde transport

**Figure 3:**
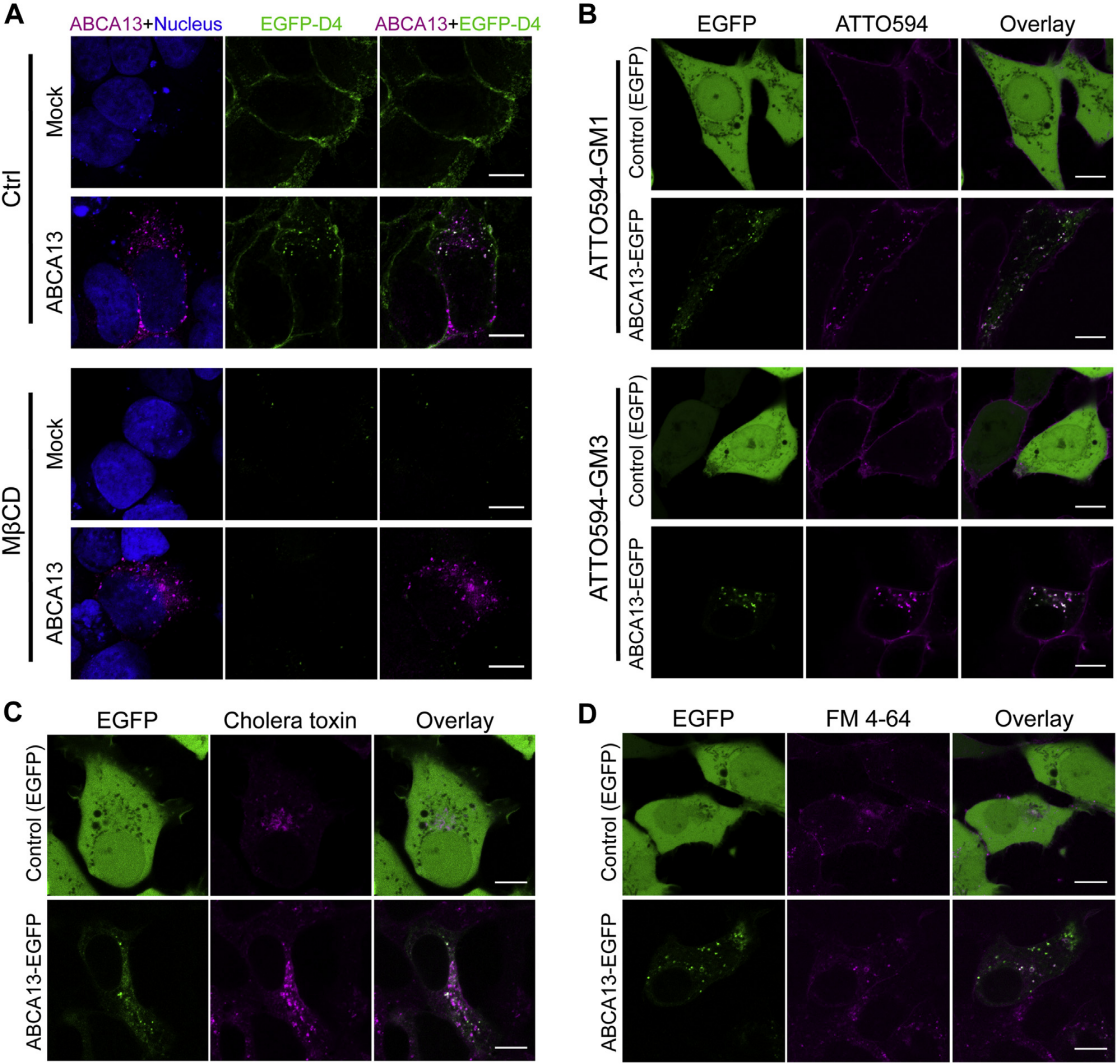
**ABCA13 mediates lipid transport from the plasma membrane to intracellular vesicles**. *A*, HEK293 cells transfected with ABCA13 were treated with or without MβCD for 10 min at 37 °C. Then, the cells were incubated with EGFP-D4 for 30 min at 37 °C and immunostained with anti-ABCA13 antibody (*magenta*) after fixation and permeabilization. Nuclei were stained with TOTO-3 (*blue*). *B* and *C*, HEK293 cells transfected with ABCA13-EGFP or EGFP (control) were incubated with ATTO594-conjugated GM1 or GM3 or with Alexa Fluor 555-conjugated cholera toxin subunit B for 30 min at 37 °C. Then, the culture medium was changed to medium without fluorescent probes, and the cells were further incubated for 30 min at 37 °C. The cells were observed using fluorescence microscopy. *D*, HEK293 cells transfected with ABCA13-EGFP or EGFP were stained with FM 4 – 64 for 30 min at 37 °C. Scale bars represent 10 μm.

Lipids are transferred between the plasma membrane and subcellular organelles (26, 27). Since the plasma membrane contains 40–90% of total cellular cholesterol (28), we hypothesized that the cholesterol accumulated in vesicles by ABCA13 was derived from the plasma membrane. To evaluate this possibility, we used EGFP-D4 to track plasma-membrane cholesterol in living cells. Incubation at 37 °C can internalize EGFP-D4, which binds to cholesterol of the exofacial leaflets of the plasma membrane, in living cells (29). Accordingly, we found EGFP-D4 was internalized and colocalized in ABCA13-vesicles in HEK293 cells (Fig. 3*A*), but not in mock transfected cells under the same conditions. Pretreatment with MβCD, which removes cholesterol from the plasma membrane (30), resulted in no binding of EGFP-D4 to the cell even though ABCA13-vesicles were detected. These results suggest that ABCA13 accelerates the internalization of cholesterol from the plasma membrane.

Because gangliosides are known to form microdomains containing cholesterol at the plasma membrane (31, 32, 33), we examined whether gangliosides such as GM1 and GM3 were also incorporated into ABCA13-vesicles. HEK293 cells transiently transfected with EGFP fused to ABCA13 at its C terminus or EGFP alone (control) were incubated at 37 °C with ATTO594-conjugated GM1 or GM3 (33), (Fig. 3*B*). ATTO594-conjugated GM1 and GM3 were substantially accumulated in ABCA13 vesicles, but their internalization was scarcely observed in control cells. Cholera toxin subunit B conjugated to Alexa Fluor 555, which binds to gangliosides (34), was also delivered to ABCA13 vesicles (Fig. 3*C*). These results suggest ABCA13 also regulates the intracellular distribution of gangliosides.

When cells were incubated with FM 4-64, a membrane-impermeant fluorescent lipid probe that is commonly used as a fluorescent reporter for endocytic vesicles and lipid trafficking (35, 36), we found FM 4-64 was localized in ABCA13 vesicles (Fig. 3*D*). The ratio of cholesterol to choline phospholipids in HEK293 cells was not altered by ABCA13 expression (Fig. S2), indicating that ABCA13 did not affect cellular cholesterol content. These results suggest that ABCA13 accelerates the internalization of plasma-membrane lipids by endocytic retrograde transport.

### Cholesterol internalization by ABCA13 requires the N-terminal region and ATP hydrolysis

**Figure 4:**
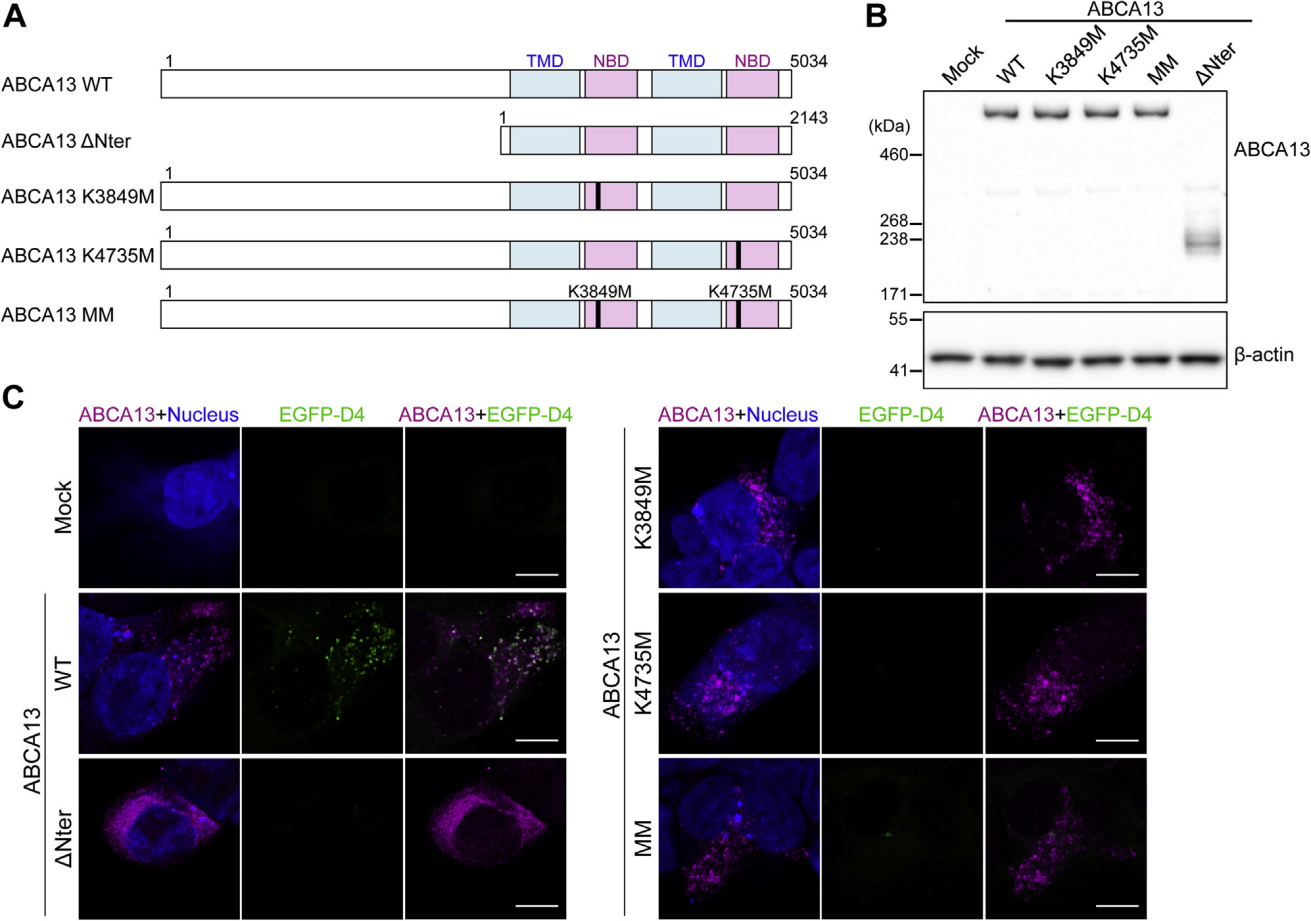
**ABCA13 requires the N-terminal region and ATP hydrolysis to trigger cholesterol accumulation**. *A*, schematic representation of the ABCA13 mutants used in this experiment. TMDs and NBDs are shown in *blue* and *red*, respectively. Point mutation positions are labeled in *black*. The numbers indicate amino acid numbering. *B*, HEK293 cells were transiently transfected with WT ABCA13 or mutants. ABCA13 expression was confirmed by western blotting. β-actin was used as a loading control. *C*, HEK293 cells transfected with WT ABCA13 or mutants were labeled with anti-ABCA13 antibody (*magenta*) and the fluorescent probe EGFP-D4 (*green*) after fixation and permeabilization. Nuclei were stained with TOTO-3 (*blue*). Scale bars represent 10 μm.

Previous studies reported that ABCA13 is also expressed as a short version lacking the long N-terminal region, starting from methionine at 2892 (5, 6). Therefore, we assessed whether the long N-terminal region is required for ABCA13 function. ABCA13 mutant without the N-terminal region (ABCA13 ΔNter, Fig. 4*A*) was generated and transfected into HEK293 cells. We confirmed that ABCA13 ΔNter was expressed as a 240 kDa protein (Fig. 4*B*) and that it showed reticular localization (Fig. 4*C*), suggesting that the N-terminal region is required for the localization of ABCA13 to intracellular vesicles.

ABC proteins couple the energy of ATP binding and hydrolysis to a variety of biological functions (3). We examined whether cholesterol internalization was dependent on ATP hydrolysis by ABCA13. We constructed three mutants: a mutant bearing one point mutation in NBD1 (ABCA13 K3849M), one in NBD2 (ABCA13 K4735M), and point mutations in both NBDs (ABCA13MM), in which conserved lysine residues in the Walker A motif crucial for ATP binding and hydrolysis were replaced by methionine (37, 38) (Fig. 4*A*). These mutant proteins were expressed at similar levels (Fig. 4*B*) and localized to the intracellular vesicles like ABCA13 WT (Fig. 4*C*). However, EGFP-D4 staining was scarcely observed with the ATP hydrolysis-deficient mutants (Fig. 4*C*). These results suggest that cholesterol internalization is dependent on ATP hydrolysis by ABCA13.

### Abca13 KO mice display significantly reduced levels of prepulse inhibition

**Figure 5:**
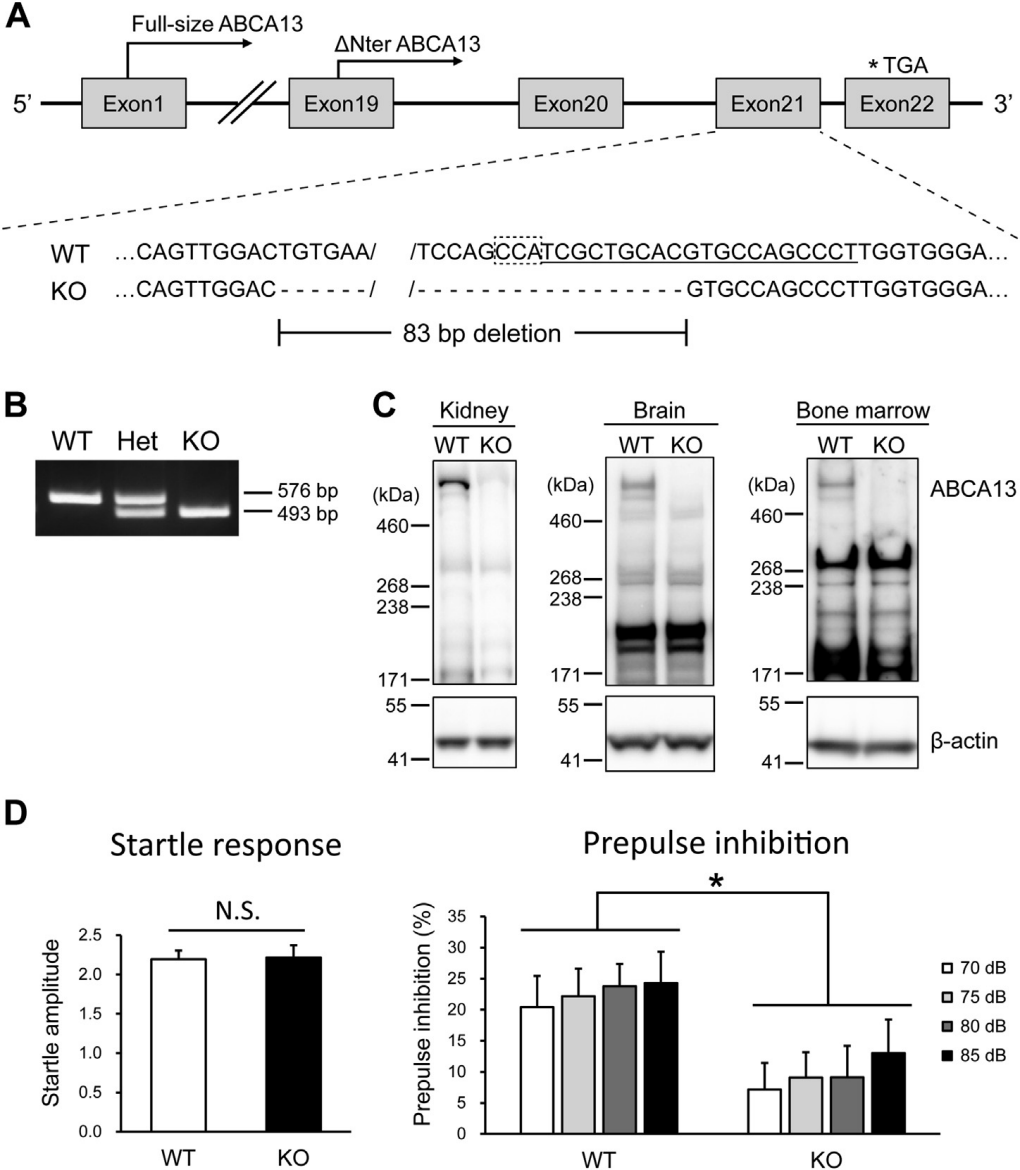
**Generation and prepulse inhibition of Abca13 KO mice.***A*, schematic representation of Abca13 KO mice generation by the CRISPR/Cas system. A schematic of the Abca13 gene locus with the genomic sequences of WT (upper) and KO (lower) mice is shown. The sgRNA sequence is underlined, and the PAM sequence is indicated by the dotted box. Exons are indicated by *boxes*, and introns are indicated by *lines*. The *asterisk* indicates the stop codon by 83 bp deletion. The translation start sites for full-size ABCA13 and ΔNter ABCA13 are indicated by the *arrows*. *B*, a representative result of genotyping PCR with genomic DNA from WT, Abca13 heterozygous KO, and homozygous KO mice. *C*, Western blot analysis of the kidney, brain, and bone marrow cells from WT and Abca13 KO mice using anti-ABCA13 antibody. β-actin was used as a loading control. *D*, acoustic startle response for 120 dB (left) and prepulse inhibition for 70–120, 75–120, 80–120, and 85–120 dB (right) in WT and Abca13 KO mice. Data are shown as means + S.E.M. (WT, n = 16; KO, n = 15). ∗*p* < 0.05 compared with WT mice.

To study the physiological role of ABCA13, we examined the effects of ABCA13 deletion on phenotypes by generating Abca13 KO mice using the CRISPR/Cas system on the genetic background of C57BL/6N strain (39, 40). To disrupt both full-size (5034 amino acids) and ΔNter (2143 amino acids) mouse Abca13, sgRNA was designed to target exon 21 of the Abca13 gene to induce frameshift mutations within the open reading frame (Fig. 5*A*). Plasmids expressing Cas9 and sgRNA against Abca13 were microinjected into zygotes of C57BL/6N mice, and Abca13 gene mutations were confirmed by DNA sequencing analysis. We created a mutant line harboring an 83 bp deletion in exon 21, leading to a premature stop codon in exon 22. The genotyping PCR products were represented in 576 bp for WT and 493 bp for KO (Fig. 5*B*). Western blotting was performed using the kidney, brain, and bone marrow to confirm the deletion of ABCA13 protein (Fig. 5*C*). A major band of ABCA13 was detected at >460 kDa in WT mice but not in KO mice. The bands detected around 240 kDa in both WT and Abca13 KO mice were considered to be nonspecific, because the frameshift mutation was introduced within the open reading frame of ΔNter ABCA13 (Fig. 5*A*) and the predicted molecular mass of ΔNter mouse ABCA13 is 240 kDa. These results indicate that the 83 bp deletion induced by the CRISPR/Cas system resulted in the deletion of ABCA13 protein.

Abca13 KO mice were born normally and seemed to have normal appearance and life span. Because it was suggested that genetic variants of human ABCA13 are related to psychiatric disorders (7), we examined Abca13 KO mice using a battery of behavioral tests including body weight measurement, open field test, light and dark transition test, elevated plus maze test, hot plate test, social interaction test, forced swim test, rotarod test, startle response/prepulse inhibition test, Y-maze test, Barnes maze test, fear conditioning test, and tail suspension test. We found no statistically significant differences in any behavioral tests (data not shown) except for the startle response/prepulse inhibition test. Prepulse inhibition, which describes the suppression of the startle reflex by a weak prepulse immediately before the startle stimulus, is a robust measure of sensorimotor gating across species, including human and rodents (41). The disruption of prepulse inhibition in patients with psychiatric disorders such as schizophrenia and bipolar disorder and in animal models of schizophrenia has been well described (41, 42, 43). In the startle response/prepulse inhibition test, no statistically significant differences between WT and Abca13 KO mice were detected in the startle response, but Abca13 KO mice displayed significantly reduced levels of prepulse inhibition (Fig. 5*D*). These results indicate that Abca13 KO mice have sensorimotor gating deficits.

### ABCA13 deletion impairs synaptic vesicle endocytosis

**Figure 6:**
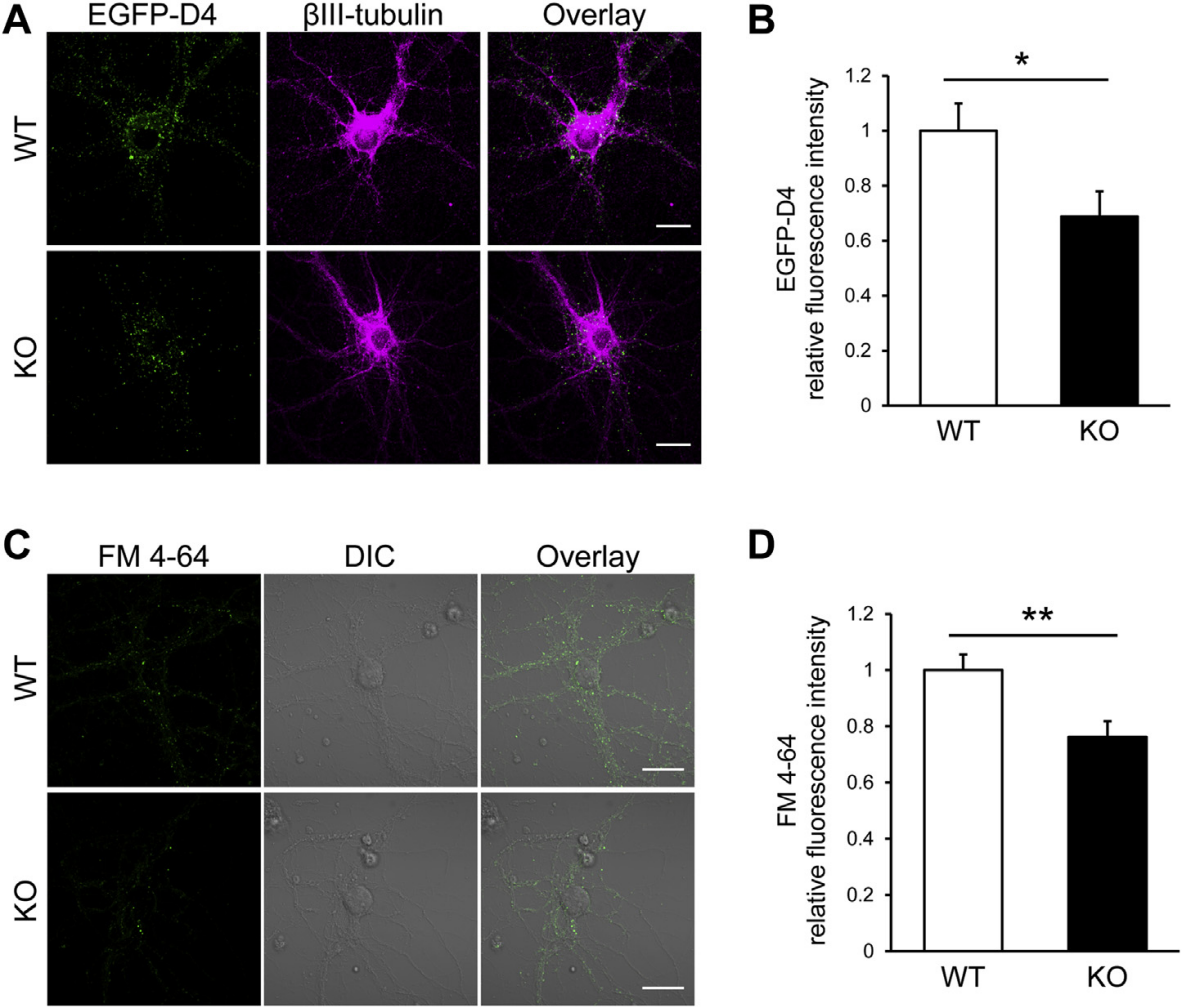
**ABCA13 deletion impairs vesicular cholesterol accumulation and synaptic vesicle endocytosis in neurons.***A*, primary mouse cortical neurons were treated with 5 mM MβCD for 5 min at 37 °C. Then, the cells were labeled with anti-βIII-tubulin antibody (*magenta*) and the fluorescent probe EGFP-D4 (*green*) after fixation and permeabilization. *B*, the relative fluorescence intensities of EGFP-D4 signals in individual cells were quantified using ImageJ and shown as means + S.E.M. (n = 30). *C*, primary mouse cortical neurons were stained with FM 4-64 for 3 min in high K+ buffer. Then, the cells were washed for 10 min in Ca^2+^-free buffer and observed using fluorescence microscopy. *D*, the relative fluorescence intensities of FM 4-64 signals in individual cells were quantified using ImageJ and shown as means + S.E.M. (n = 30). Scale bars represent 20 μm. ∗*p* < 0.05, ∗∗*p* < 0.01.

To examine the effects of ABCA13 deletion on intracellular cholesterol distribution in neurons, primary mouse cortical neurons at 14 days *in vitro* were pretreated with MβCD and stained with EGFP-D4 (Fig. 6*A*). Intracellular EGFP-D4 signals in Abca13 KO neurons were decreased compared with WT neurons. Quantitative analysis of EGFP-D4 staining revealed that the fluorescence intensity of EGFP-D4 was significantly lower in Abca13 KO neurons (Fig. 6*B*). These results indicate that ABCA13 dysfunction leads to decreased accumulation of vesicular cholesterol in cortical neurons.

Previous study demonstrated the importance of a high vesicular sterol concentration for synaptic vesicle endocytosis (44). Therefore, we investigated whether ABCA13 deletion impairs the endocytosis of synaptic vesicles using FM dye. Primary cortical neurons at 14 days *in vitro* were incubated with FM 4-64 in high K+ solution to load the synaptic vesicles with FM dye during depolarizing stimulation (45). FM 4-64 was internalized in neurons during synaptic vesicle endocytosis, and FM 4-64 signals in Abca13 KO neurons were decreased compared with WT neurons (Fig. 6*C*). Quantitative analysis showed that uptake of FM 4-64 was significantly reduced in Abca13 KO neurons (Fig. 6*D*). These results suggest that ABCA13 deletion impairs synaptic vesicle endocytosis in cortical neurons.

### Rare coding variants of ABCA13 related to psychiatric disorders impair the function of ABCA13

**Figure 7:**
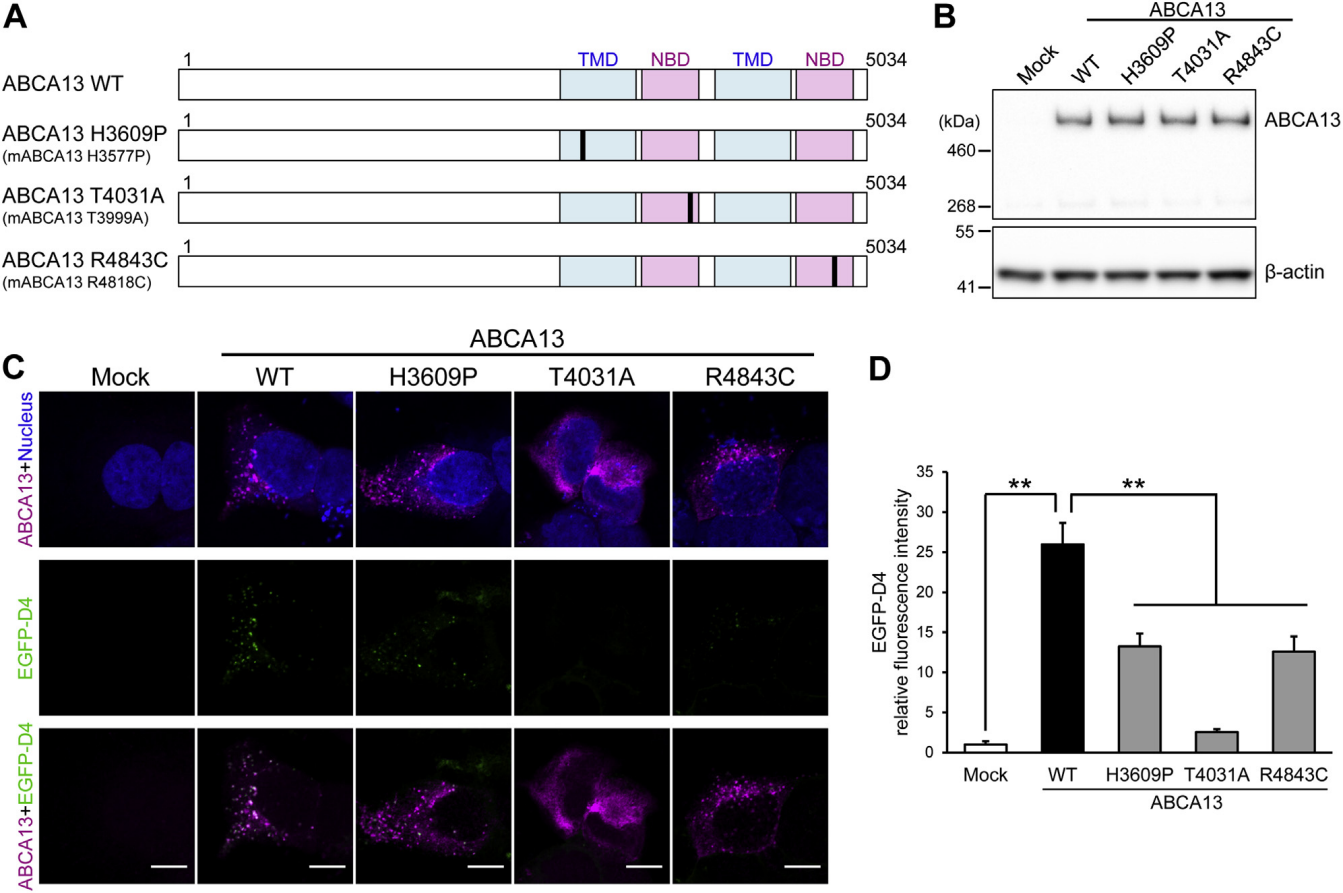
**ABCA13 mutations associated with psychiatric disorders impair the subcellular localization or function of ABCA13.***A*, schematic representation of the ABCA13 mutants used in this experiment. TMDs and NBDs are shown in *blue* and *red*, respectively. Point mutation positions are labeled in *black*. The numbers indicate amino acid numbering. *B*, HEK293 cells were transfected with WT ABCA13 or mutants. ABCA13 expression was confirmed by western blotting. β-actin was used as a loading control. *C*, HEK293 cells transfected with WT ABCA13 or mutants were labeled with anti-ABCA13 antibody (*magenta*) and the fluorescent probe EGFP-D4 (*green*) after fixation and permeabilization. Nuclei were stained with TOTO-3 (*blue*). Scale bars represent 10 μm. *D*, the relative fluorescence intensities of EGFP-D4 signals in individual cells were quantified using ImageJ and shown as means + S.E.M. (n = 50). ∗∗*p* < 0.01 compared with WT ABCA13.

Rare genetic variants of human ABCA13 were reported to increase the susceptibility to schizophrenia, bipolar disorder, and major depression (7). Especially, three missense mutations (H3609P, T4031A, and T4550A) are significantly more frequent in bipolar cases than controls and one missense mutation (R4843C) is more common in schizophrenia cases. To examine the effects of mutations associated with psychiatric disorders on the function of ABCA13, H3609P, T4031A and R4843C were introduced into the corresponding amino acid residues of mouse ABCA13 (H3577P, T3999A, and R4818C, respectively, Fig. 7*A*). Because T4550 in human ABCA13 is not conserved in mouse ABCA13, T4550A mutation was not examined in this study. WT and mutant proteins were expressed at similar levels in HEK293 cells (Fig. 7*B*). T4031A mutant was not localized to intracellular vesicles, and reticular localization was observed (Fig. 7*C*). In the case of H3609P and R4843C mutants, intracellular cholesterol accumulation in the vesicles was decreased compared with WT, but their vesicular localization was normal. Quantitative image analysis of EGFP-D4 staining revealed that cholesterol accumulation was significantly lower in cells with ABCA13 mutants (Fig. 7*D*). A scatter plot of the relative fluorescence intensity of ABCA13 and of EGFP-D4 in individual cells shows the effect of the ABCA13 mutants on the EGFP-D4 signal (Fig. S3*A*). However, the ABCA13 signal was unchanged (Fig. S3*B*). These results suggest that mutations associated with psychiatric disorders impair either the subcellular localization or function of ABCA13.

## Discussion

Previous studies have suggested that rare coding variants of human ABCA13 contribute to the risk of schizophrenia, bipolar disorder, and major depression (7). However, little is known about the molecular functions of ABCA13 or their association with psychiatric disorders. In this study, we found that ABCA13 accelerated the internalization of cholesterol and gangliosides by endocytic retrograde transport. In addition, we found that Abca13 KO mice exhibited deficits of prepulse inhibition and that ABCA13 deletion impaired synaptic vesicle endocytosis. Finally, mutations of ABCA13 associated with psychiatric disorders impaired the protein's subcellular localization and function.

Lipids are involved in many important physiological processes including cell signaling, growth, migration, and differentiation, and the intracellular distribution of lipids is tightly controlled to cellular organization and functions (26, 46, 47, 48). It has been reported that ABCA1, which exports intracellular excess cholesterol to generate the high-density lipoprotein, regulates the cholesterol synthesis pathway by facilitating retrograde cholesterol transport from the plasma membrane to the endoplasmic reticulum (49). In this study, we found that ABCA13 accelerates the internalization of cholesterol and gangliosides from the plasma membrane to intracellular vesicles. These results suggest that ABCA13 plays an important role for cellular lipid homeostasis by endocytic retrograde lipid transport. However, the mechanisms for ABCA13-mediated retrograde lipid transport remain to be elucidated. Interestingly, previous studies have shown that early endosomes containing ABCA1 shuttle between the plasma membrane and intracellular endocytic compartments even though ABCA1 is predominantly localized in the plasma membrane (50, 51). This endocytic pathway contributes to the intracellular trafficking of substrate lipids. Given these findings, there is a possibility that ABCA13 localized at the plasma membrane forms endocytic vesicles containing high levels of cholesterol and gangliosides to mediate lipid trafficking.

The size of human ABCA13 gene is controversial. One study indicated the gene spans 450 kb and encodes an exceptionally large ABC protein of 5058 amino acids (4). Another study, however, argues that the gene spans only about 367 kb of genomic DNA and codes for a protein of no more than 2323 amino acids (5). Our study provides new findings that ABCA13 with the long N-terminal region is mainly expressed in mice and regulates lipid internalization to intracellular vesicles. While our results do not exclude the existence of shorter ABCA13, they do provide convincing evidence for the physiological importance of longer ABCA13. The N-terminal region does not have homology to any known human proteins or contain any well-conserved domains. We speculate that it regulates ABCA13 interactions with other proteins for the proper localization and cholesterol internalization.

Deficits in prepulse inhibition are observed in patients and animal models of various psychiatric disorders including schizophrenia and qualify as a robust endophenotype for genetic evaluation (52). The impaired prepulse inhibition in Abca13 KO mice we observed suggests that the dysfunction of ABCA13 is related to the pathophysiology of psychiatric disorders. Because Abca13 KO mice exhibited a normal startle response, physical and sensory abnormalities such as hearing loss do not seem to contribute to the impairment of prepulse inhibition. Furthermore, we found that the body weight and behavioral phenotypes except for the prepulse inhibition of Abca13 KO mice were normal, indicating that the dysfunction of prepulse inhibition was not due to changes in body weight, motor function, activity, or anxiety levels. These findings suggest that ABCA13 specifically affects the brain function regulating sensorimotor gating in mice.

Our study indicates that ABCA13 causes cholesterol accumulation in intracellular vesicles and accelerates the endocytosis of synaptic vesicle in cortical neurons. Cholesterol is enriched in the membrane of synaptic vesicles (53), and the levels of cholesterol in synaptic vesicles have a strong impact on synaptic functions including biogenesis and exocytosis of synaptic vesicles (54). Our observations are consistent with the previous study demonstrating that high vesicular sterol content is required for synaptic vesicle recycling (44). Mutations in several genes regulating synaptic vesicle trafficking are linked to psychiatric disorders including schizophrenia and bipolar disorder (55, 56). Given these findings, dysregulation of synaptic vesicle trafficking by ABCA13 dysfunction likely contributes to the pathophysiology of psychiatric disorders. It might be necessary to examine neuronal activity and neurotransmitter release in Abca13 KO mice in addition to the localization of ABCA13 in mouse brain to link impaired synaptic vesicle endocytosis more closely to psychiatric disorders. While the ratio of cholesterol to choline phospholipids in HEK293 cells was not altered by ABCA13 expression (Fig. S2), we cannot exclude the possibility that ABCA13 dysfunction affects cholesterol content or ganglioside composition in specific neurons. We would like to examine this possibility in the near future, when the antibody detecting mouse endogenous ABCA13 is available.

The three missense mutations in ABCA13 we investigated (H3609P, T4031A, and R4843C) were selected because they increase the susceptibility to schizophrenia and bipolar disorder (7). We found that these mutations impaired the subcellular localization or function of ABCA13. H3609 is predicted to be located near the membrane-spanning helix (4), suggesting H3609P mutation might affect the structure of the transmembrane helices, leading to ABCA13 dysfunction. On the other hand, T4031 and R4843 are located in the NBDs, which are crucial for ATP binding and hydrolysis. T4031 is located downstream of the Walker B motif of NBD1 and conserved in the ABCA subfamily (57). The substitution of the corresponding amino acid in ABCA1 (T1088A) severely impairs the protein's subcellular localization and function (57). Consistent with that report, our results showed that T4031A in ABCA13 impairs the subcellular localization and cholesterol accumulation in vesicles, suggesting that this amino acid residue is crucial for protein folding of NBD1. R4843 resides between the Walker A and B motifs of NBD2. Unlike T4031A, R4843C in ABCA13 did not impair subcellular localization but impaired cholesterol trafficking. Notably, substitution of the corresponding amino acid in ABCA1 (K2031C) has no effect on that protein's function (57), suggesting that this substitution has effects specific to ABCA13.

To summarize, we found that full-size ABCA13 is expressed *in vivo* and accelerates the internalization of cholesterol and gangliosides from the plasma membrane to intracellular vesicles. ABCA13 gene mutations associated with psychiatric disorders impaired the subcellular localization and function of ABCA13. Together, these findings provide new insights into the biological function of ABCA13. In addition, Abca13 KO mice exhibited deficits in prepulse inhibition and ABCA13 deletion impaired synaptic vesicle endocytosis, suggesting that a loss of function of ABCA13 is associated with the pathophysiology of psychiatric disorders and that the established Abca13 KO mice might make a useful animal model of psychiatric disorders. Further studies on the function of ABCA13 might lead to the development of novel therapeutic strategies for psychiatric disorders.

## Experimental procedures

### Materials

To immunostain mouse ABCA13, rabbit anti-ABCA13 antibody was generated against the C terminus of mouse ABCA13 (57). To immunostain human ABCA13, rabbit polyclonal anti-ABCA13 antibody (#HPA063601) was purchased from Sigma-Aldrich. For immunoblotting, rabbit polyclonal anti-ABCA13 antibody (#LS-C373172) was purchased from LifeSpan BioSciences. Mouse anti-GFP antibody was purchased from Santa Cruz Biotechnology. Anti-β-tubulin antibody, methyl-beta-cyclodextrin (MβCD), and filipin complex were purchased from Sigma-Aldrich. Mouse anti-β actin antibody was purchased from Abcam. Mouse antineuron specific βIII-tubulin antibody was purchased from Merck Millipore. Cholera toxin subunit B conjugated to Alexa Fluor 555, FM 4-64, propidium iodide, and TOTO-3 were purchased from Invitrogen. ATTO594-conjugated GM1 and GM3 were synthesized as reported (33).

### Cloning and plasmid construction

The cDNA encoding the open reading frame of mouse Abca13 gene (NCBI accession No. NM_178259) was amplified by PCR using mouse kidney cDNA as a template. The cDNA was inserted into pcDNA3.1/Hygro(+). EGFP was inserted into the C terminus of Abca13. The ΔN-terminal deletion mutant of Abca13, in which 2891 residues were absent from the N-terminus, was generated by PCR and subcloned into pcDNA3.1/Hygro(+). All other Abca13 mutants were generated by site-directed mutagenesis using the In-Fusion HD Cloning Kit (Clontech). The expression vector for EGFP-θ toxin domain 4 (D4) (25) was a kind gift from Dr Kobayashi (Universite de Strasbourg). The plasmids expressing Cas9 and single guide RNA (sgRNA) against Abca13 were prepared by ligating oligos (5ʹ-AGGGCTGGCACGTGCAGCGA-3ʹ) into the BbsI site of pX330-U6-Chimeric_BB-CBh-hSpCas9 as described previously (39).

### Cell culture and transfection

HEK293 cells were cultured in Dulbecco's modified Eagle's medium (DMEM) supplemented with 10% fetal bovine serum at 37 °C in a humidified atmosphere containing 5% CO_2_. The cells were seeded at a density of 6.5 × 10^4^ cells/cm^2^ on glass coverslips coated with poly-L-lysine (Sigma-Aldrich) or 12-well plates. After 24 h, the cells were transfected with the expression vectors using Lipofectamine LTX Reagent with PLUS Reagent (Invitrogen) and incubated for 48 h. U2OS cells were cultured in McCoy's 5A medium supplemented with 10% fetal bovine serum. The cells were plated at a density of 1.8 × 10^4^ cells/cm^2^ on glass coverslips coated with fibronectin (Sigma-Aldrich). After 24 h, stealth RNAi siRNA against Abca13 (5ʹ-GGAGTACTTGCTGGCACCATCTGAA-3ʹ) or stealth RNAi siRNA negative control (Invitrogen) was transfected into the cells using Lipofectamine RNAiMAX Transfection Reagent (Invitrogen). The cells were then incubated for 48 h. Primary neuronal cultures were prepared from the cerebral cortex of neonatal mice (postnatal day 0). Neonatal mice were killed by decapitation. Skulls were removed and cerebral cortices were dissected from mouse brain in ice-cold HBSS, and cells were isolated using Neuron Dissociation Solutions (FUJIFILM Wako Pure Chemical Corporation) according to the manufacturer's instructions. The cells were suspended in Neurobasal medium supplemented with B-27 supplement (Gibco) and GlutaMAX (Gibco) and seeded at a density of 2.6 × 10^4^ cells/cm^2^ on glass coverslips coated with poly-D-lysine (Sigma-Aldrich). The cells were cultured for 14 days at 37 °C in a humidified atmosphere containing 5% CO_2_.

### Immunostaining

Cells on glass coverslips were fixed with 4% paraformaldehyde for 15 min, permeabilized with 0.25% Triton X-100 for 5 min, and then incubated with 10% goat serum for 1 h at room temperature to reduce nonspecific binding of the antibodies. The cells were incubated with the primary antibodies overnight at 4 °C and then with the secondary antibodies conjugated to fluorescent Alexa Fluor dyes and TOTO-3 or propidium iodide for 1 h at room temperature. Observations were made using an LSM 700 laser scanning confocal microscope (Carl Zeiss) or an IX83 inverted microscope (Olympus).

### Filipin staining

Cells on glass coverslips were fixed with 4% paraformaldehyde for 15 min at room temperature, permeabilized with 0.25% Triton X-100 for 5 min on ice, and then incubated with 0.05 mg/ml filipin/PBS for 30 min at room temperature. Observations were made using the IX83.

### EGFP-D4 purification

*E. coli* strain BL21(DE3) was used to overexpress EGFP-D4. After induction with IPTG, *E. coli* cells were harvested and resuspended in PBS. The cell suspension was sonicated and centrifuged, and EGFP-D4 was purified from the supernatant using Profinity IMAC Ni-Charged Resin (BIO-RAD). EGFP-D4 was concentrated using an Amicon Ultra-0.5 Centrifugal Filter Unit 3K (Merck). An equal volume of glycerol was added, and the sample was stored at −30 °C.

### Plasma membrane staining by EGFP-D4

Cells on glass coverslips were treated with 5 mM MβCD in 0.02% bovine serum albumin (BSA)/DMEM for 10 min at 37 °C. Then the cells were washed with HBSS and treated with 5 μg/ml EGFP-D4 in 0.1% BSA/HBSS for 30 min at 37 °C. After washing, the cells were fixed with 4% paraformaldehyde for 15 min at room temperature. Observations were made using the LSM 700.

### Intracellular staining by EGFP-D4 and quantification

Cells were fixed with 4% paraformaldehyde for 10 min at room temperature, permeabilized with 0.25% Triton X-100 for 5 min on ice, and then incubated with 5 μg/ml EGFP-D4 in 0.1% BSA/HBSS for 30 min at room temperature. After washing, the cells were refixed with 4% paraformaldehyde for 10 min. The cells were photographed blindly with the LSM 700. The fluorescence intensity in individual cells was analyzed using NIH ImageJ software.

### Live cell imaging and quantification

Cells were incubated with 0.5 μg/ml Alexa Fluor 555-conjugated cholera toxin subunit B or 1 μM ATTO594-conjugated GM1 or GM3 for 30 min at 37 °C. Then the culture medium was changed to 0.02% BSA/DMEM without fluorescent probes, and the cells were further incubated for 30 min at 37 °C. After washing, the cells were observed using the LSM 700. For FM dye uptake experiments in HEK293 cells, cells were stained with 5 μg/ml FM 4-64 in 0.02% BSA/HBSS for 30 min at 37 °C. After washing, the cells were observed using the LSM 700. For cultured neurons, cells were incubated with 5 μg/ml FM 4-64 for 3 min in high K+ buffer (60 mM KCl, 67 mM NaCl, 30 mM glucose, 2 mM CaCl_2_, 2 mM MgCl_2_, and 25 mM HEPES, pH 7.4). After FM 4-64 loading, the cells were washed for 10 min in Ca_2_+-free buffer (2 mM KCl, 125 mM NaCl, 30 mM glucose, 2 mM MgCl_2_, and 25 mM HEPES, pH 7.4) and photographed blindly with the LSM 700. The fluorescence intensity in individual cells was analyzed using ImageJ.

### Measurement of lipid content

HEK293 cells were transfected with the expression vectors and incubated for 48 h. Then, lipids were extracted from the cells with chloroform/methanol (2:1). The lipid-containing solution was dried and resuspended in 2-propanol, and the levels of cholesterol and choline phospholipids were determined by colorimetric assay kits purchased from FUJIFILM Wako Pure Chemical Corporation (435-35801 for free cholesterol and 433-36201 for choline phospholipids).

### Western blotting

Cultured cells or tissues were lysed with RIPA lysis buffer, and equal amounts of protein were reduced by 50 mM dithiothreitol, separated by SDS-PAGE, transferred to polyvinylidene difluoride membrane, and probed with the primary antibodies. Secondary antibodies conjugated to horseradish peroxidase were detected using an Immunostar LD/Zeta (FUJIFILM Wako Pure Chemical Corporation) and Ez-Capture (ATTO).

### Generation and maintenance of Abca13 KO mice

Abca13 KO mice were generated using the CRISPR/Cas system as described previously (39). sgRNA against mouse Abca13 was designed using CRISPRdirect design software (58). Briefly, plasmids expressing Cas9 and sgRNA were microinjected into the zygotes of C57BL/6N mice. Mutations on Abca13 were confirmed by DNA sequencing at FASMAC. Homozygous KO mice were born from a heterozygous intercross and used for the analysis in parallel with age- and sex-matched WT littermates as a control group. All mice were genotyped 2 weeks after birth and housed according to their gender after weaning. Mice were kept under standard conditions of feeding and lightening (12 h light/dark cycle). All animal experiments were conducted in accordance with institutional policies following approval from the Animal Experimentation Committee of Institute for integrated Cell-Material Sciences and the Graduate School of Agriculture, Kyoto University.

### Genotyping

The genotypes were confirmed by PCR using genomic DNA, KOD FX Neo (TOYOBO), and specific primers (forward, 5ʹ-AACTAGCAACTGGGCTCTGG-3ʹ, and reverse, 5ʹ-CATCTGACAGCAAAGGCTGC-3ʹ). PCR was performed as follows: 2 min at 94 °C, and 30 cycles of 10 s at 98 °C, and 60 s at 68 °C.

### Tissue sampling

Mice were euthanized, and the kidney, brain, and bone marrow cells were collected and immediately frozen with liquid nitrogen. Collected samples were stored at –80 °C until protein extraction.

### Startle response/prepulse inhibition test

A startle reflex measurement system (O'Hara & Co) was used to measure the startle response and prepulse inhibition. Mice were placed in a plastic cylinder for 10 min. The response to a startle noise (white noise, 40 msec) was recorded for 140 msec starting with the onset of the prepulse stimulus. The peak startle amplitude recorded during the 140 msec was used as the dependent variable. The background noise level in each chamber was 70 dB. The intensity of the startle stimulus was 120 dB. The prepulse sound was presented 100 msec before the startle stimulus, and its intensity was 70, 75, 80, or 85 dB. Four combinations of prepulse and startle stimuli were used (70–120, 75–120, 80–120, and 85–120 dB). Each was presented in pseudorandom order. The trials were separated by an average interval of 15 sec (range 10–30 sec).

### Statistical analysis

Statistical analysis of the fluorescence intensities and cholesterol content was performed using unpaired Student's *t*-test or one-way ANOVA followed by Dunnett's test. Startle response/prepulse inhibition data were analyzed by unpaired Student's *t*-test or two-way repeated measures ANOVA.

## Data availability

All data are contained within the article.

## Conflict of interest

The authors declare that they have no conflicts of interest with the contents of this article.
