# Supplementary material for: ABCA13 dysfunction associated with psychiatric disorders causes impaired cholesterol trafficking
Source: J Biol Chem. 2020 Dec 16;296:100166. doi: 10.1074/jbc.RA120.015997 (PMC7948424; doi:10.1074/jbc.RA120.015997)
Supplement: Supplementary file 1 — Figures S1 to S3 [file mmc1.pdf]

Figure S1. ABCA13 is expressed in U2Os cells.

U2OS cells, a human osteosarcoma cell line, were or were not transfected with a negative control or ABCA13-targeted siRNA. Cells were immunostained with anti-ABCA13 antibody (green). Nuclei were stained with TOTO-3 (blue). Scale bars represent 50 μm.

Figure S2. ABCA13 does not alter cellular cholesterol content.

Lipids were extracted from HEK293 cells transiently transfected with ABCA13, and the levels of cholesterol and choline phospholipids were measured by colorimetric enzyme assay. The ratio of cholesterol to choline phospholipids are shown as means + S.E.M. (n = 4).

Figure S3. Expression levels of ABCA13 WT and mutants.

(A) Scatter plot of the relative fluorescence intensities of ABCA13 signals and of EGFP-D4 signals in individual cells transfected with ABCA13 WT or mutants. (B) The relative fluorescence intensities of ABCA13 signals in cells transfected with ABCA13 WT or mutants are shown as means + S.E.M. (n = 50).

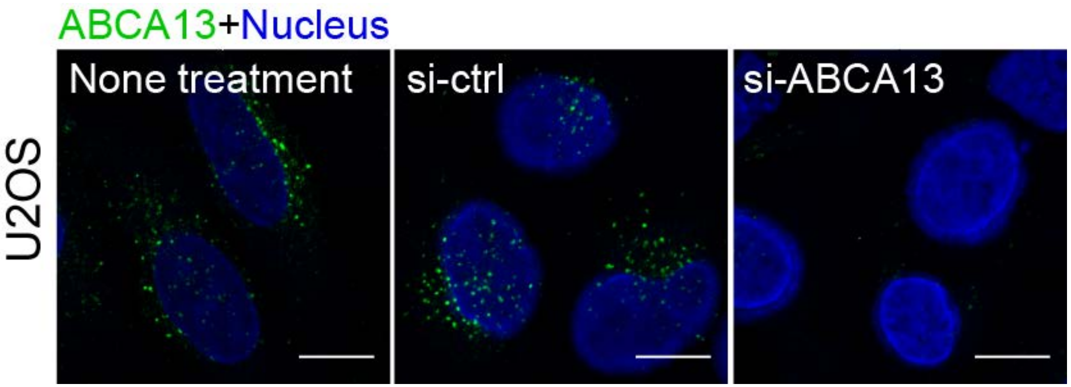

Fig. S1 Nakato, M. et al.

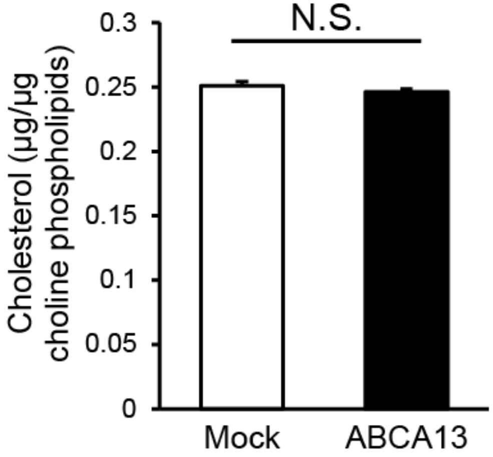

Fig. S2 Nakato, M. et al.

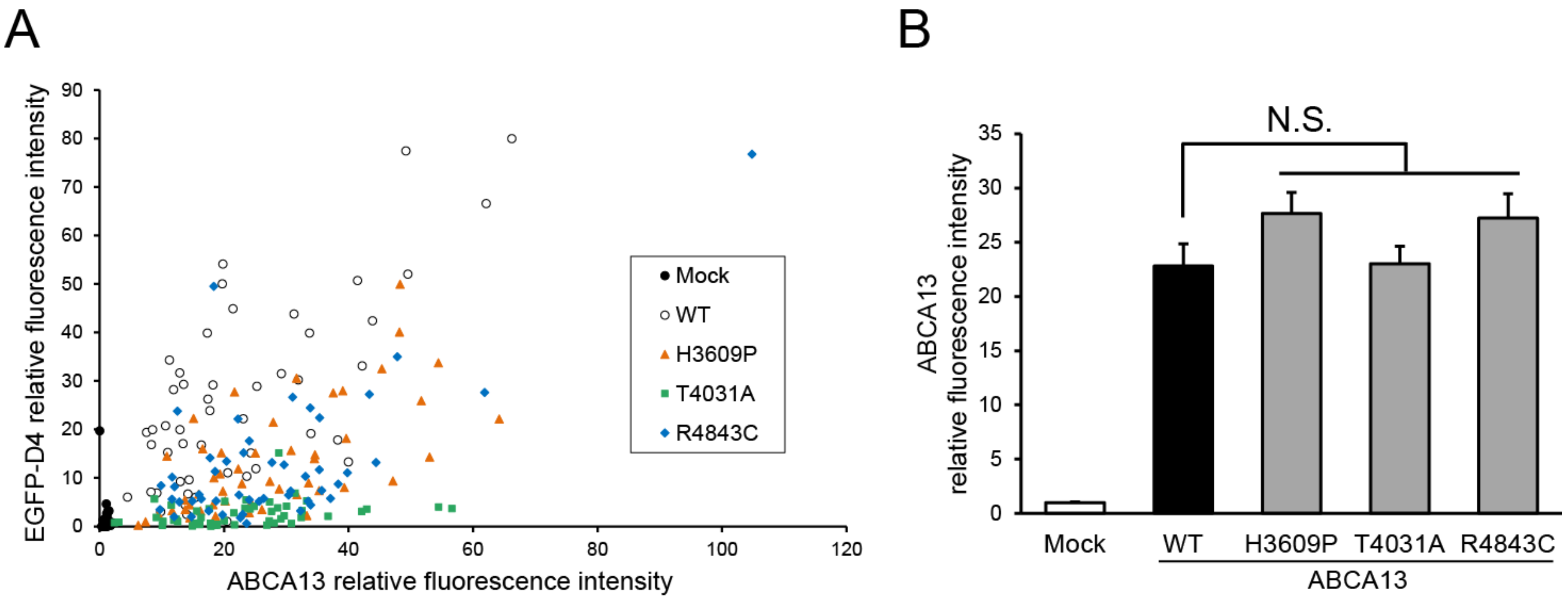

Fig. S3 Nakato, M. et al.
